# Assessment of the Soft-Tissue Seal at the Interface between the Base of the Fixed Denture Pontic and the Oral Mucosa

**DOI:** 10.3390/ma14143997

**Published:** 2021-07-16

**Authors:** Ikiru Atsuta, Ikue Narimatsu, Taichiro Morimoto, Chi-Hsiang Cheng, Kiyoshi Koyano, Yasunori Ayukawa

**Affiliations:** 1 Division of Advanced Dental Devices and Therapeutics, Faculty of Dental Science, Kyushu University, Fukuoka 812-8582, Japan; koyano@dent.kyushu-u.ac.jp; 2 Section of Implant and Rehabilitative Dentistry, Division of Oral Rehabilitation, Faculty of Dental Science, Kyushu University, Fukuoka 812-8582, Japan; narimatu.i@dent.kyushu-u.ac.jp (I.N.); teikeishoudds@gmail.com (C.-H.C.); ayukawa@dent.kyushu-u.ac.jp (Y.A.); 3 Morimoto Dental Clinic, Fukuoka 813-0013, Japan; taichiro_morimoto@yahoo.co.jp

**Keywords:** fixed denture, epithelial seal, oral mucosa, adhesion molecule

## Abstract

Fixed dentures (bridges) are often selected as a treatment option for a defective prosthesis. In this study, we assess the contact condition between the base of the pontic and oral mucosa, and examine the effect of prosthetic preparation and material biocompatibility. The molars were removed and replaced with experimental implants with a free-end type bridge superstructure after one week. In Experiment 1, we assessed different types of prosthetic pre-treatment: (1) the untreated control group (Con: mucosa recovering from the tooth extraction); (2) the laser irradiation group (Las: mucosa recovering after the damage caused by a CO_2_ laser); and (3) the tooth extraction group (Ext: mucosa recovering immediately after the teeth extraction). In Experiment 2, five materials (titanium, zirconia, porcelain, gold-platinum alloy, and self-curing resin) were placed at the base of the bridge pontic. Four weeks after the placement of the bridge, the mucosa adjacent to the pontic base was histologically analyzed. In Experiment 1, the Con and Las groups exhibited no formation of an epithelial sealing structure on the pontic base. In the Ext group, adherent epithelium was observed. In Experiment 2, the sealing properties at the pontic interface were superior for titanium and the zirconia compared with those made of porcelain or gold-platinum alloy. In the resin group, a clear delay in epithelial healing was observed.

## 1. Introduction

Fixed denture (bridge) treatment is indispensable as a prosthetic treatment option for dental defects in dental practice [1]. The abutment teeth on both sides of the defect are prepared and joined to a pontic that replaces the missing tooth to form a bridge. In most cases, the base of the pontic is brought into horizontal surface contact with the oral mucosa to achieve optimal esthetics and comfort [2]. The pontic is designed to make firm contact with the oral mucosa. In the upper anterior region, an ovate pontic that presses firmly against the gingiva immediately after tooth extraction may be selected [3]. The health of the oral mucosa in contact with the base of the pontic is an important issue. In cases where the prosthetic device has been detached, clear redness is often observed in the oral mucosa that was in contact with the pontic. It is unclear whether this is simply an inflammation of the oral mucosa under difficult cleaning conditions or the pontic is adhering to the mucosal epithelium. It is also unclear whether the results will differ depending on the material used for the pontic base.

It is known that the peri-implant tissue around titanium or zirconia implants has a biological border with an adhesive and binding structure formed by epithelial tissue and connective tissue [4,5]. When the soft tissue wound heals after implantation, a temporary adhesive structure is thought to be generated by epithelial cells coming into contact with the titanium surface [6]. In other words, the repair of connective tissue occurs as a normal healing process of the oral mucosa immediately after tooth extraction and epithelial cells move horizontally over the connective tissue to cover the wound [7]. However, because the implant body impedes the horizontal growth and migration of the epithelial cells after implant insertion, they cannot be connected from left to right and thus begin to proliferate deeply along the surface of the implant body and turn back at a certain depth. It is reported that this flow creates the epithelial attachment structure around the implant [7,8].

Unlike the healing mucosa around the implant, the bridge pontic generally is in contact with healthy oral mucosa, thus it would seem to be impossible to obtain epithelial attachment. However, the pressure of the pontic base against the wound occurring immediately after tooth extraction is similar to the case of adherent epithelium formation around the implant apart due to the vertical healing being replaced by horizontal healing. In such a case, a similar adhesion structure may be formed. No studies have yet focused on this phenomenon, though.

In this study, the adhesion strength between the base of the pontic and soft tissue was evaluated based on the expression level of laminin (an epithelial adhesion-related protein) and on the invasion distance of horseradish peroxidase (HRP). As this experimental method has been used in a previous paper as a method for evaluating soft-tissue sealing properties [4,9,10], its reliability is high. It is well known that laminin secreted from epithelial cells forms a structure called a hemidesmosome that adheres to the extracellular matrix via heparan sulfate or a similar substance [11]. Therefore, it is thought that thick linear deposition on the enamel surface at the boundary of mucosa around the natural teeth and on the titanium surface around the implant is an indicator of sealing by epithelial tissue [7,12].

Depending on whether or not the base of the bridge pontic adheres to the oral mucosa, the results obtained in this study answer questions about bridge maintenance such as whether to clean the pontic base daily. In addition, the results obtained provide criteria for choosing the material to be used for the pontic base. The answers to these questions will be useful for clinicians in future practice.

## 2. Materials and Methods

### 2.1. Experimental Bridge Model

**Figure 1:**
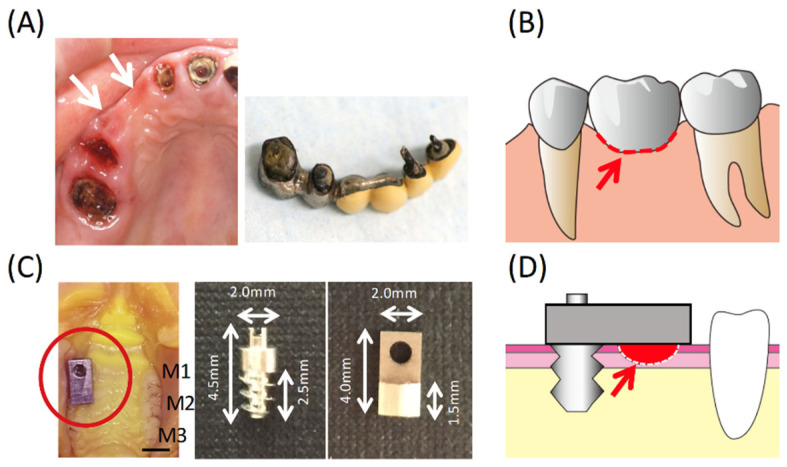
(**A**) The oral mucosa (white arrows) in contact with the bridge pontic base and the detached bridge pontic base. (**B**) Schematic diagram of the bridge area (the red dot and arrow indicate the focus area of this study). (**C**) Photographs of the experimental implants and the implant in the rat oral cavity with no evidence of inflammation. Bar = 2 mm. (**D**) Scheme of the experimental model and the observed area on the base of the bridge pontic.

**Figure 2:**
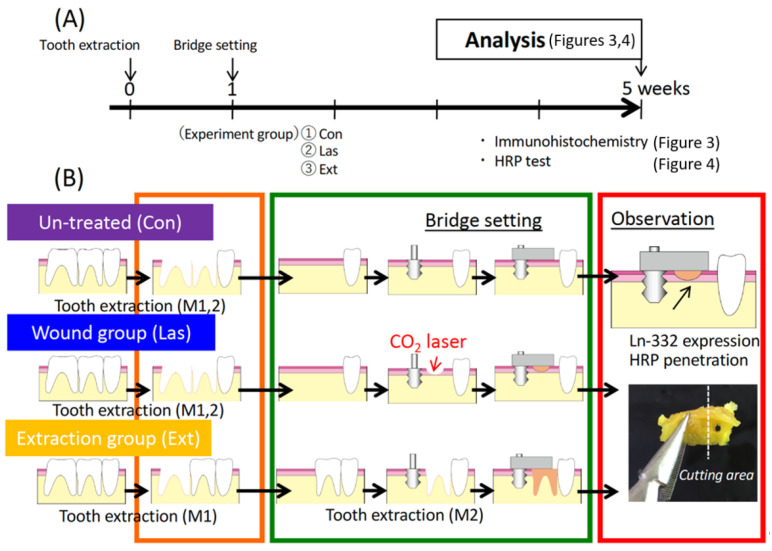
Experimental bridge model and experimental protocol. (**A**) Experimental protocol for the in vivo study. (**B**) Experimental groups; Con: mucosa healing after tooth extraction; Las: mucosa treated with a CO_2_ laser after tooth extraction; and Ext: mucosa after a tooth extraction.

**Figure 3:**
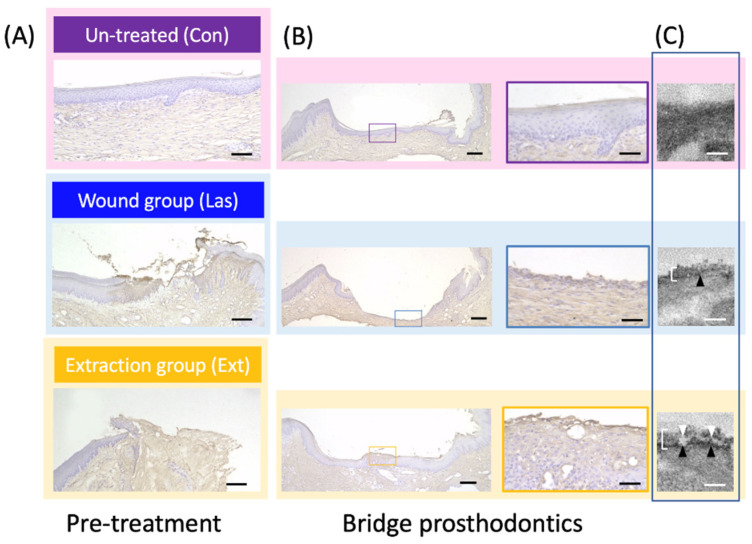
Image of laminin-332 immunostaining of the oral mucosa in contact with the bridge pontic base. (**A**) Light microscope image of the oral mucosa immediately before the placement of the bridge after each treatment. Bar = 200 µm. (**B**) Microscope image of the oral mucosa in contact with the base of the pontic at 4 weeks after bridge placement. Bar = 100 µm. (**C**) Electron microscope image presenting samples immune-stained with laminin-332. The arrow-heads indicate the normal appearance with a dual layer of Ln-332 staining representing the lamina densa (black) and lamina lucida (white). The white line indicates HD-like structures. Bar = 100 nm.

**Figure 4:**
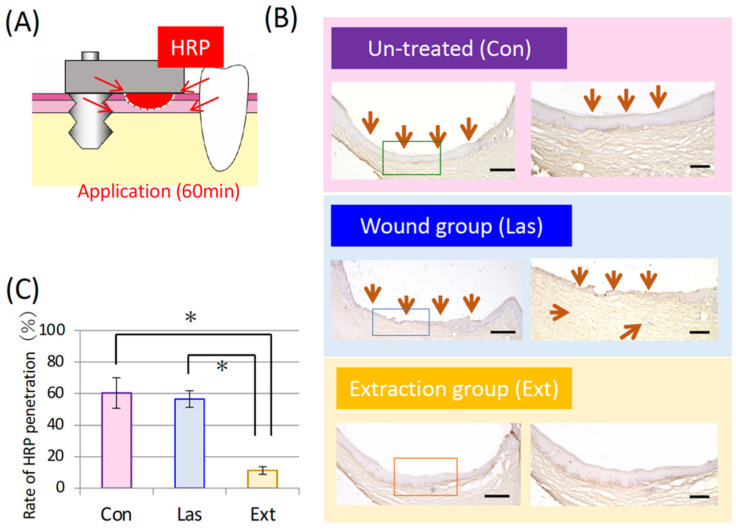
Adhesion between the bridge pontic base and the oral mucosa. (**A**) Schematic diagram of an experiment in which horseradish peroxidase (HRP) was added from the boundary between the pontic base and oral mucosa. (**B**) Microscope image of chemically stained HRP in each group (low magnification on the left and magnified image on the right). Bar = 100 µm. (**C**) Graph presenting the proportion of individuals in which HRP penetrated the center of the base of the pontic. Each bar represents the mean ± SD. One-way ANOVA with Scheffe’s post-hoc test; * *p* < 0.05 between the indicated groups.

**Figure 5:**
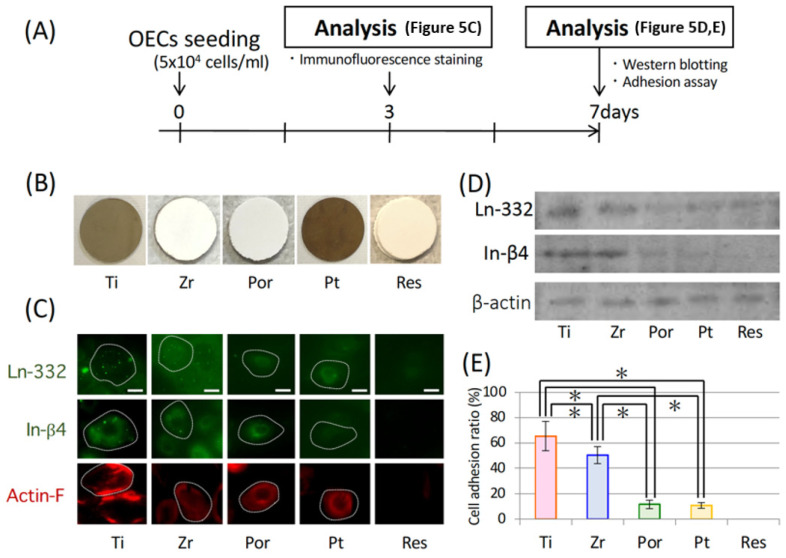
Epithelial cell adhesion on the five materials. (**A**) Experimental protocol for the in vitro study. (**B**) Titanium (Ti), Zirconia (Zr), porcelain (Por), gold-platinum alloy (Pt), and self-curing resin (Res) plates were prepared for culture study as the experimental group. (**C**) Localization of the adhesion-related proteins in the cells on Ti, Zr, Por, Pt, and Res. Bar = 15 µm. (**D**) Western blotting data. (**E**) Epithelial cell adhesion ratio. Each bar represents the mean ± SD. One-way ANOVA with Scheffe’s post-hoc test; * *p* < 0.05 between the indicated groups.

**Figure 6:**
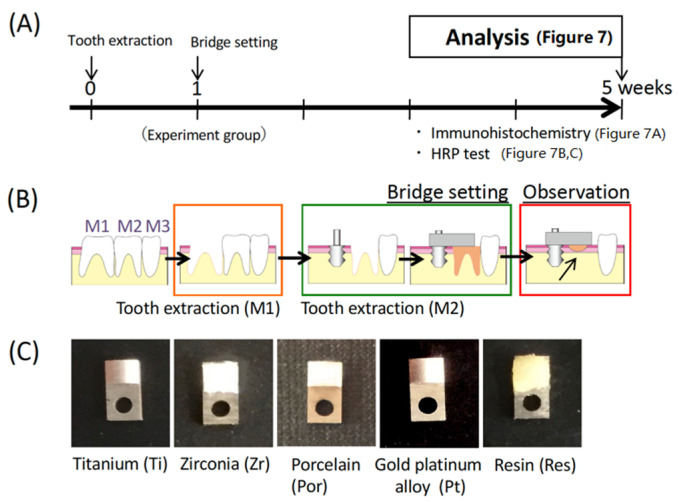
Selecting the most effective material for the bridge pontic base. (**A**) Experimental protocol. (**B**) Experimental model. (**C**) The five materials used in the study.

**Figure 7:**
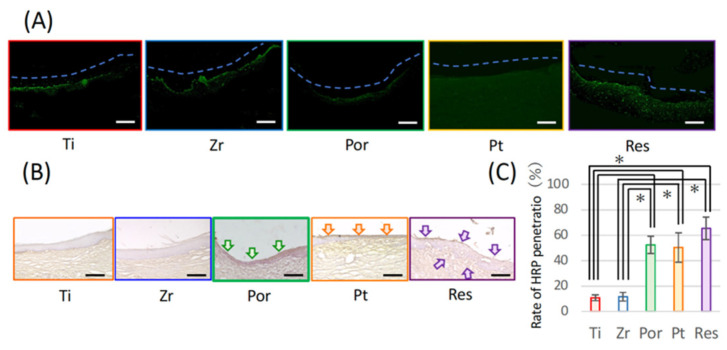
Image of the oral mucosa in contact with the base of bridge pontics built from the five materials. (**A**) Image of fluorescent immunostaining with laminin-332. Bar = 100 μm. (**B**) Light microscope image of chemically stained horseradish peroxidase (HRP) in each group. Bar = 150 μm. (**C**) Graph illustrating the rate at which HRP penetrated the center of the base of the pontic. Each bar represents the mean ± SD. One-way ANOVA with Scheffe’s post-hoc test; * *p* < 0.05 between the indicated groups.

The following model was created assuming the clinical state illustrated in Figure 1A,B consisting of a screw-type pure titanium (Ti) implant with two pieces, a screw part (length 4.5 mm and diameter 2 mm) implanted into alveolar bone, and a plate part (length 4 mm and width 2 mm) (Sky Blue, Fukuoka, Japan) covering the oral mucosa (Figure 1C). The screw part of this implant was modeled from previously described designs [13,14] and the plate part was designed according to the space formed by the removal of two rat molars. The surface topographies were similar to the experimental plates used in our culture experiments. Additionally, only the titanium in Figure 2, Figure 3 and Figure 4 was in contact with the oral mucosa, while in Figure 5, Figure 6 and Figure 7, zirconia (Zr), gold-platinum alloy (Pt), porcelain (Por), and self-curing resin (Res; Unifast II, GC, Tokyo, Japan) were attached to the titanium plate for evaluation.

### 2.2. Animals

Rats received care following the guidelines established by Kyushu University (Fukuoka, Japan, approval number: A25-240-0). Experimental implantation was performed as in previous reports [7,15]. Briefly, 6-week old Wistar rats (in total 50 males (5 rats per group for immune-stain or HRP experiment); 120–150 g) had maxillary right first molars extracted under systemic anesthesia followed by placement of the experimental implants for the bridge abutment.

### 2.3. Experimental Groups

As shown in Figure 2 and Figure 3 groups were prepared for Experiment 1. As a control 1 week after extraction of the maxillary right first and second molars, the base of the pontic material was pressed against the mucosa during epithelial healing (Con group). A carbon dioxide laser was used to apply a shallow wound to the epithelium without bleeding and then pressure was applied by the implant plate (Las group). The oral mucosa in contact with the base of the pontic was damaged to the level of the connective tissue immediately after tooth extraction and pressure was applied to the bleeding wound (Ext group). Note that Figure 6 and Figure 7 refer only to the Ext group.

### 2.4. Immunohistochemistry (Light Microscopy)

At 4 weeks after implantation, the rats were euthanized. The oral mucosa was removed from the maxillary bone and sections were cut on the coronal plane using a cryostat (−20 °C). These sections were immunohistochemically stained with rabbit anti-Ln-332 (Chemicon International., Temecula, CA, USA) and biotinylated anti-rabbit IgG (Sigma, St. Louis, MO, USA), and then visualized with a diaminobenzidine (DAB) staining kit (Vector Laboratories, Burlingame, CA, USA) according to the method outlined in a previous paper [16].

### 2.5. Immunohistochemistry (Electron Microscopy)

All samples were cut into 10-μm-thick sections and stained with anti-Ln-332 antibody, biotinylated antibody, and DAB-H_2_O_2_ solution. Next, the samples were fixed with 0.1% OsO_4_ and immersed in Quetol 653 resin (Nisshin EM, Tokyo, Japan). These sections were observed by electron microscopy (JEOL, Tokyo, Japan) as reported previously [7].

### 2.6. Horseradish Peroxidase (HRP) Test

HRP (50 mg/mL; Sigma, St. Louis, MO, USA), which has a molecular weight (40,000 Da) similar to lipopolysaccharide (LPS), was impregnated into a cotton ball and topically applied for 60 min to the gingival margin around the bridge pontics of 35 rats at 4 weeks after implantation. Then, frozen sections around the bridge were prepared and stained by the DAB method according to the method outlined in a previous paper [9,17]. In this study, the distance of HRP penetration was measured in a horizontal direction. The percentage of the HRP-positive bottom of the 2 mm wide pontic was measured.

### 2.7. Culture Experiments

Ti plates (99.9 mass%, Sky Blue, Fukuoka, Japan), Zr (Sky Blue, Fukuoka, Japan), Por, Pt, and Res plates were prepared for culture study as outlined in a previous paper [10,18]. Oral epithelial cells were isolated from the oral mucosa of 4-day old Wistar rats and cultured in a defined keratinocyte serum-free medium (DK-SFM; Thermo Fisher Scientific, Waltham, MA, USA) on each plate (Figure 5A,B).

### 2.8. Immune or Chemical-Fluorescence Staining for Adhesion Proteins

Oral epithelial cells cultured on the plates were fixed and then stained with anti-rat Ln-332, anti-rat integrin (In)-β4, or anti-rat plectin polyclonal antibodies (Chemicon International, Temecula, CA, USA). Additionally, TRITC-conjugated phalloidin (Sigma Chemical Co., Balcatta, WA, USA; 1:100) was used for actin filament staining.

### 2.9. Western Blotting

Proteins were separated by 7.5% SDS-PAGE, transferred to PVDF membranes (Bio-Rad Laboratories, Hercules, CA, USA), and immunoblotted with anti-Ln-332 or anti-In-β4 antibodies. Antibody-bound bands were visualized by using Enhanced ChemiLuminescence (ECL; GE Healthcare, Boston, MA, USA).

### 2.10. Adhesion Assay

As adhesion assays, the adhering oral epithelial cells on the five materials were counted before and after using a rotary shaker (NX-20; Nissin, Tokyo, Japan) [19]. The percentage of remaining adherent cells was defined as the adhesive power of the cells.

### 2.11. Statistical Analysis

Our experiment used 5 samples in each group and a priori Shapiro–Wilk test was performed to test for normality. One-way analysis of variance (ANOVA) with Scheffe’s post-hoc test was performed. Values of *p* < 0.05 were considered statistically significant. Data are indicated as the mean ± standard deviation (SD).

## 3. Results

### 3.1. Condition of the Oral Mucosa at the Bridge Pontic Base

Figure 3A illustrates the oral mucosa in contact with the base of the pontic. In the Con group, mucosal healing after the tooth extraction was observed. In the Las group that had only the CO_2_ laser application to the oral mucosa with complete healing after tooth extraction, only a part of the epithelial tissue and connective tissue was missing. In the Ext group, both the soft and hard tissue were damaged immediately after tooth extraction.

A bridge was placed on the oral mucosa in the above groups and the morphology of the mucosa 4 weeks later is presented in Figure 3B. In the Ext group, the expression of Ln-332 was observed in a line on the surface in contact with the base of the pontic in the light microscope image and a positive cell layer around 100-μm thick was observed as a band in the electron microscope image. In the Las group, epithelial healing was rather delayed and no positive layer of Ln-332 was observed. In contrast, in the Con group the mucosal condition had hardly changed from before the bridge attachment and no Ln-332 expression was observed. No major difference was observed in the condition of the connective tissue in any of the groups.

### 3.2. Epithelial Adhesion to the Base of the Bridge Pontic

To confirm whether adhesion occurred between the pontic base and the epithelium, HRP, which simulates LPS, was absorbed from around the pontic and the depth of penetration was evaluated (Figure 4A). In the Ext group, HRP hardly reached the base of the pontic. In contrast, both the Las group and Con group exhibited abundant penetration of HRP along the base of the pontic (Figure 4B). Figure 4C presents the HRP penetration rate in a graph. In 60% or more of the Las and Con groups, the HRP reached the center of the base of the pontic but almost no permeation was observed in the Ext group.

### 3.3. Influence of Materials on Epithelial Cells

The dynamics of the epithelial cells were observed for the five materials used for the base of the pontic in the in vivo experiment. The epithelial cells in the Ti and Zr groups exhibited strong expression of Ln-332 and in-β4 which was observed by fluorescent immunostaining. Cytoskeleton development was demonstrated by the fluorescent staining of actin filaments. Compared with the Por, Pt, and Res groups, the intracellular skeleton in the Ti and Zr groups was thicker and more continuous (Figure 5C). In fact, western blotting demonstrated that the expression levels of adhesion-related proteins were significantly higher in the Ti and Zr groups (Figure 5D). The adhesion strength of the epithelial cells in the Ti and Zr groups was significantly higher than that of the Por and Pt groups (Figure 5E). However, in the case of the Res group, no cell adhesion was observed in the first place and measurement was not possible in these experiments.

### 3.4. Influence of Material Used for the Bridge Pontic Base

Immediately after tooth extraction, an experimental implant with a free-end bridge was placed in the socket (Figure 6B) and the base of the pontic was composed of one of the five materials (Figure 6C). Ln-332 expression was linearly observed at the interface between the pontic base and the mucosa in the titanium (Ti) and zirconia (Zr) groups but no positive layer was observed for porcelain (Por) or gold platinum (Pt). In contrast, a wide-ranging positive reaction was observed in the resin (Res) group (Figure 7A). In the experiment investigating HRP infiltration, almost no infiltration was observed in the Ti and Zr groups but infiltration was observed up to the center of the base in the Por and Pt groups. In the Res group, the infiltration also reached the central part of the pontic and further penetrated into the connective tissue (Figure 7B). This is shown graphically in Figure 7C.

## 4. Discussion

There are many types of pontic structures in bridge devices but in most cases their bases are firmly in contact with the mucosal epithelium [2]. Once placed, the bridge superstructure cannot be removed and it is difficult to observe the condition of the oral mucosa just under the pontic. However, as depicted in Figure 1A, when the prosthetic device is detached the oral mucosa just under the pontic base is often observed to be reddened. Whether the redness observed is an inflammatory reaction caused by pontic pressure or whether it is an epithelium-like adhesive structure that has formed on the pontic is impossible to distinguish with the naked eye. Whether the oral mucosa is inflamed or has acquired adhesion with the material is considered to have a great influence on how maintenance should be conducted and may be of interest to a clinician. Therefore, as presented in Figure 1D, a model imitating the relationship between the pontic base and the oral mucosa was created.

As our laboratory has been conducting studies using a rat model with titanium mini-implants since 2002, we selected this experimental model with a cantilever bridge [4,7,20]. We also created three groups that mimic clinical situations (Figure 2B). One group represented the most common clinical situation in which the pontic base was brought into contact with untreated normal mucosa (Mucosa group (Con)). The second group represented the situation in which the mucosa had only epithelial defects via a CO_2_ laser wound but with no bleeding (Wound group (Las)) because the CO_2_ laser can provide a hemostatic effect for wounds [21]. Considering the importance of blood flow, one group represented the situation in which the pontic is placed in contact with the extraction socket immediately after tooth extraction (Extraction group (Ext)).

As demonstrated in Figure 3, only the Ext group that had substantial damage to the connective tissue immediately after tooth extraction exhibited adhesiveness to the titanium pontic base; this seems to be closely related to the blood supply. Ln-332, used in the present study to estimate epithelial adhesion to the pontic base, has demonstrated in many previous studies to be an indicator of epithelial adhesion to enamel or titanium [6,22]. This extracellular protein is an adhesion-related protein expressed by epithelial cells but it binds to integrin-α6β4 on the cell membrane surface via proteoglycans such as heparan sulfate, forming hemidesmosomes and adhering to the extracellular matrix [9,11]. Titanium, which is not part of the organism, can be treated in the same way as an extracellular matrix [23,24,25]. Additionally, integrin is known as a mediator for actin filaments that act as a cytoskeleton via plectin to enhance cell adhesion [26,27]. That is, the expression level of laminin can be used as a criterion for evaluating the adhesive strength for in vivo experiments. For in vitro experiments, the expression level of laminin and its related proteins forming its complex during cell adhesion can be evaluated, as well the development of a cytoskeleton [28]. Therefore, the continuous deposition observed with light microscopy and the thick accumulation observed in the electron microscope images can indicate strong adhesion between epithelial cells and the extracellular matrix [4]. However, as described above, the presence of proteoglycan in the blood is essential for the formation of this laminin-centered basement membrane. Moreover, as many growth factors are expressed in wound healing [29,30], the epithelial adhesion to the material may have been improved by the influence of an insulin-like growth factor-1 [27]. As a result, as depicted in Figure 3, thick and mature Ln-332 was observed only in the Ext group with blood supply as a result of the tooth extraction, thin deposits were observed in the Las group and almost no Ln-332 was observed in the Con group. These findings suggest that the pontic base had adhesion to the epithelium only in the Ext group. We also conducted an experiment, as depicted in Figure 4A, using HRP to confirm the strength of the soft-tissue seal. As described in the materials and methods section, HRP is a substance with a molecular weight of 40,000 that imitates LPS and the amount of HRP that permeates the base of the pontic is used to evaluate the resistance to LPS produced by bacteria and other substances. In other words, deep penetration into the body indicates a weak blockade [9,10].

As depicted in Figure 4B, the Ext group had complete sealing, whereas the Las group allowed invasion into the connective tissue. The graph in Figure 4C illustrates the measured value of HRP penetration along the base of the pontic and demonstrates that the weakest blockade is in the Con group. However, because the histology indicates a completely keratinized layer at the boundary with titanium, there seems to be little harm to the organization. These findings indicate that large mucosal lesions with bleeding may acquire epithelial adhesion to the pontic base during the healing process but small lesions with only epithelial damage result in decreased bio-sealing.

What kind of materials can provide effective sealing between the pontic base and the oral mucosa? In clinical practice, gold-platinum alloy and porcelain are often used as bridge pontic bases. Additionally, in recent years, titanium and zirconia have often been used; thus, in this study, experiments were conducted using these four materials including polymerization resin that is used as the material for temporary crowns. To select the most suitable dental material for achieving epithelial adhesion, we can refer to studies that demonstrate which material adheres most effectively to the gingiva in oral implants. As depicted in Figure 5, oral mucosa-derived epithelial cells found on each material were cultured and the expression of adhesion-related proteins and adhesions were compared. A stronger expression of adhesion-related proteins for epithelium was observed in the titanium and zirconia group when compared with the gold-platinum alloy and porcelain groups. This result was also confirmed by the results of the in vivo study. That is, it is presumed that the adhesiveness of titanium or zirconia is high and the adhesiveness of gold-platinum alloy is low for achieving a seal with the epithelial tissue. Figure 7 presents results that are consistent with this prediction. As depicted in Figure 7A, the adhesion protein Ln-332 is expressed in titanium and zirconia, and HRP invasion was clearly inhibited in both these groups (Figure 7B). However, HRP intrusion was observed along the base of the pontic in the gold-platinum alloy, porcelain groups, and in the connective tissue in the resin group.

Our findings of strong adhesion of epithelial tissue to titanium and zirconia and weak adhesion to gold-platinum alloy are consistent with the findings of Furuhashi et al. [31]. It is thought that the reason for this substantial difference is due to the fact that gold-platinum alloy is a highly ion-eluted material compared with the stability of titanium and zirconia [32,33]. Lastly, the porcelain in this clinical trial had undergone a glazing treatment and the surface after glazing can become rough [34,35]. It is known that a rough surface reduces the adhesion of epithelial cells when compared with a smooth surface. This is why porcelain seems to have low epithelial adhesion and sealing properties. Additionally, many studies have reported that unpolymerized organic solvents continue to be released for several days from the resin material on pontics [36,37]. It is thought that these solvents exert a strong toxicity on the cells and this was reflected in both the in vitro and in vivo data. In addition, the surface of the material was rough and inhibited the adhesion of epithelial cells. However, in this study, the pressure and area of the pontic base applied to the oral mucosa could not be controlled and the results may vary depending on these conditions. Our immune-histochemical observation revealed the expression of laminin-332 and bocking to HRP at the interface, indicating active adhesion of epithelial tissue to Ti and Zr, and also indicating weaker adhesion to the epithelium to the other materials. However, because we solely focused on the epithelialization and adhesion in this study, future research should be conducted with new data to evaluate inflammation and foreign body response.

## 5. Conclusions

In conclusion, the pontic base of a bridge can adhere to the oral mucosa. When the connective tissue is invasively damaged by tooth extraction, epithelial tissue adhesion to the pontic can be obtained. The material used for the base of the pontic is optimally titanium or zirconia because gold-platinum alloy, resin, and porcelain do not promote epithelial adhesion. Thus, in the clinical setting, proper selection of prosthetic preparation and materials is required to control the relationship between the bridge pontic base and the mucosa.

## Data Availability

The data presented in this study are available on request from the corresponding author.
